# Information Extraction From FDA Drug Labeling to Enhance Product-Specific Guidance Assessment Using Natural Language Processing

**DOI:** 10.3389/frma.2021.670006

**Published:** 2021-06-10

**Authors:** Yiwen Shi, Ping Ren, Yi Zhang, Xiajing Gong, Meng Hu, Hualou Liang

**Affiliations:** ^1^ College of Computing and Informatics, Drexel University, Philadelphia, PA, United States; ^2^ Office of Research and Standards, Office of Generic Drugs, Center for Drug Eval uation and Research, United States Food and Drug Administration, Silver Spring, MD, United States; ^3^ School of Biomedical Engineering, Science and Health Systems, Drexel University, Philadelphia, PA, United States

**Keywords:** information extraction, NLP, FDA drug labels, BERT, product specific guidance

## Abstract

Towards the objectives of the UnitedStates Food and Drug Administration (FDA) generic drug science and research program, it is of vital importance in developing product-specific guidances (PSGs) with recommendations that can facilitate and guide generic product development. To generate a PSG, the assessor needs to retrieve supportive information about the drug product of interest, including from the drug labeling, which contain comprehensive information about drug products and instructions to physicians on how to use the products for treatment. Currently, although there are many drug labeling data resources, none of them including those developed by the FDA (e.g., Drugs@FDA) can cover all the FDA-approved drug products. Furthermore, these resources, housed in various locations, are often in forms that are not compatible or interoperable with each other. Therefore, there is a great demand for retrieving useful information from a large number of textual documents from different data resources to support an effective PSG development. To meet the needs, we developed a Natural Language Processing (NLP) pipeline by integrating multiple disparate publicly available data resources to extract drug product information with minimal human intervention. We provided a case study for identifying food effect information to illustrate how a machine learning model is employed to achieve accurate paragraph labeling. We showed that the pre-trained Bidirectional Encoder Representations from Transformers (BERT) model is able to outperform the traditional machine learning techniques, setting a new state-of-the-art for labelling food effect paragraphs from drug labeling and approved drug products datasets.

## Introduction

Product-Specific Guidance^1^ (PSG) represents the United States Food and Drug Administration (FDA)’s current thinking on the best approaches for demonstrating bioequivalence between a test product and the reference product. The development of product-specific guidances (PSGs) intends to facilitate generic drug product development, and Abbreviated New Drug Application (ANDA) submission and approval, ultimately promote safe, effective, affordable generic drugs to the public in the United States. During the Generic Drug User Fee Amendments II (GDUFA II), FDA is committed to issuing PSGs for 90 percent of non-complex new chemical entity New Drug Applications that are approved on or after October 1, 2017, at least 2 years prior to the earliest lawful ANDA filing date^2^. This commitment, in addition to the demands of developing PSGs for complex drug products, calls for an enhanced PSG developing process. To answer this challenge, one of the solutions is to identify and automate the labor-intensive works during the PSG development. To generate a PSG, FDA staff usually need to take extensive efforts (e.g., 50% efforts for a regular immediate release solid oral dosage form drug product) to collect relevant information from multiple data resources to a PSG review template, e.g., extracting the information related to boxed warning, indication, dosage and administration, clinical pharmacology and pharmacokinetics including absorption, distribution, metabolism, excretion, food effect (ADMEF) from drug labeling as well as the reference listed drug and reference standard (RLD/RS) information from the Orange Book^3^. If this part of the PSG development work can be automatically accomplished by a well-designed data analytics tool, the PSG developers can have more time and effort to focus on the human intelligence-required work.

Natural language processing (NLP) has been increasingly used with a specific focus on text mining and information extraction in drug labeling. For example, Fung et al. (2013) extracted information from the indications section of the drug labeling from DailyMed to encode drug-indication pairs, whereas Bisgin et al. (2011) extracted three labeling sections (Boxed Warning, Warnings and Precautions, Adverse Reactions) from DailyMed to group drugs by topics that are associated with the same safety concerns and therapeutic uses. However, most of the previous work focused only on a single data source (for review, see, e.g. Fang et al., 2016). Since none of the data sources can cover all the drug products, retrieving drug labeling information from multiple sources is needed in PSG developing process. The data sources used in our PSG developing process include Orange Book, Drugs@FDA,^4^ DailyMed^5^ and DrugBank^6^, which are described as follows.

### Data Sources

#### Orange Book

Orange Book, formally known as the Approved Drug Products with Therapeutic Equivalence Evaluations, is considered to be the authoritative source of information in the United States on the therapeutic equivalence of FDA approved drug products. It includes currently marketed prescription drug products approved by the FDA through new drug applications (NDAs) and abbreviated new drug applications (ANDAs) with different dosage forms (Center for Drug Evaluation and Research 2020). It also selects the reference standard (RS) which an applicant seeking approval of an ANDA must use in conducting an *in vivo* bioequivalence study required for approval. In this paper, we used Orange Book as the baseline for the FDA application number.

#### Drugs@FDA

Drugs@FDA is a publicly available resource, which includes the majority of drug labeling, approval letters, reviews, and other information for FDA-approved drug products for human use provided by the FDA. It contains prescription brand-name drug products, over-the-counter brand-name drug products, generic drug products, and therapeutic biological products.

#### DailyMed

DailyMed is a free drug information resource provided by the United States. National Library of Medicine (NLM) that consists of digitized versions of drug labeling as submitted to the FDA. It is the official provider of the FDA labeling information (package inserts). The documents published use the Health Level Seven (HL7) version 3 Structured Product Labeling (SPL) standard, which specifies various drug label sections (Schadow 2005, 7). It uses Logical Observation Identifiers Names and Codes (LOINC) to link sections and subsections of human prescription drug and biological product labeling.

#### DrugBank

DrugBank is a richly annotated resource that combines detailed drug data with comprehensive drug target and drug action information provided by the University of Alberta and the Metabolomics Innovation Center (Wishart et al., 2008). It contains FDA-approved small molecule and biologics drugs with extensive food-drug and drug-drug interactions as well as ADMET (absorption, distribution, metabolism, excretion, and toxicity) information (Knox et al., 2011).

As a proof of concept, in this study, we proposed an information extraction pipeline using NLP and machine learning to provide an automatic data-processing workflow to extract, annotate and integrate drug labeling data from multiple publicly available data sources with minimal human intervention for PSG development. However, mining the drug labeling from multiple sources is challenging, since these data sources, including those developed by the FDA, with the various data formats and access methods, are often not easily available to inform the PSG development process.

To address the challenge of interoperation with multiple data sources, our pipeline provided examples to access data via different methods, parse data in various data formats, and unify identification scheme. At the same time, there are complex scenarios where machine learning models are essential to achieve accurate information extraction. We provided a case study for labelling food effect paragraphs (Food Effect vs. Non-Food Effect) as an example to illustrate how we addressed the scenarios when keyword detection and regular expression were not adequate. In our case study, we leveraged the pre-trained BERT (Bidirectional Encoder Representations from Transformers) model to identify food effect paragraphs from the drug labeling. The BERT (Devlin et al., 2019) is a ground-breaking unsupervised NLP model, which has been trained with huge general language datasets, such as Wikipedia Corpus, and can be fine-tuned on usually small datasets for specific language tasks to achieve the state-of-the-art performance on many NLP tasks. Therefore, in this work, instead of training a new model from scratch, we took advantage of the pre-trained BERT model for labeling food effect paragraphs to obtain better performance. We showed that the BERT-based model is able to outperform the traditional machine learning techniques for identifying food effect-related paragraphs on drug labeling datasets. It is expected that our developed information extraction pipeline will save a great amount of time and effort so that the PSG developers can devote more time to the human-intelligence-required work.

The software package for data collection, preprocessing, and the model training and validation for labeling food effect paragraphs will be provided at https://github.com/Yiwen-Shi/drug-labeling-extraction.

## Methods

We collected drug product information from multiple publicly available data sources, including Orange Book, Drugs@FDA, DailyMed, and DrugBank. The raw data varied widely in format, from well-structured XML files to highly irregular free-text PDF files. To create a structured dataset to facilitate the assessment process for PSG development, we attempted to convert various data formats into a structured data frame in four steps. First, we collected raw data from either requesting RESTful (representational state transfer) API (application program interface) or downloading the full database whenever the API is not available. Second, we parsed raw data in various data formats into structured data records. Third, we mapped different identification schemes to the FDA application number, which is a unique six-digit number assigned to a drug product by the FDA. Last, we kept the latest version if multiple versions of drug labeling existed.

We provided a case study for labeling food effect paragraphs, in which a pre-trained BERT model was finetuned on a small set of annotated labels. We showed that the BERT-based model is able to outperform the traditional machine learning techniques for food effect paragraph labeling on drug label datasets.

### Data Processing

**FIGURE 1:**
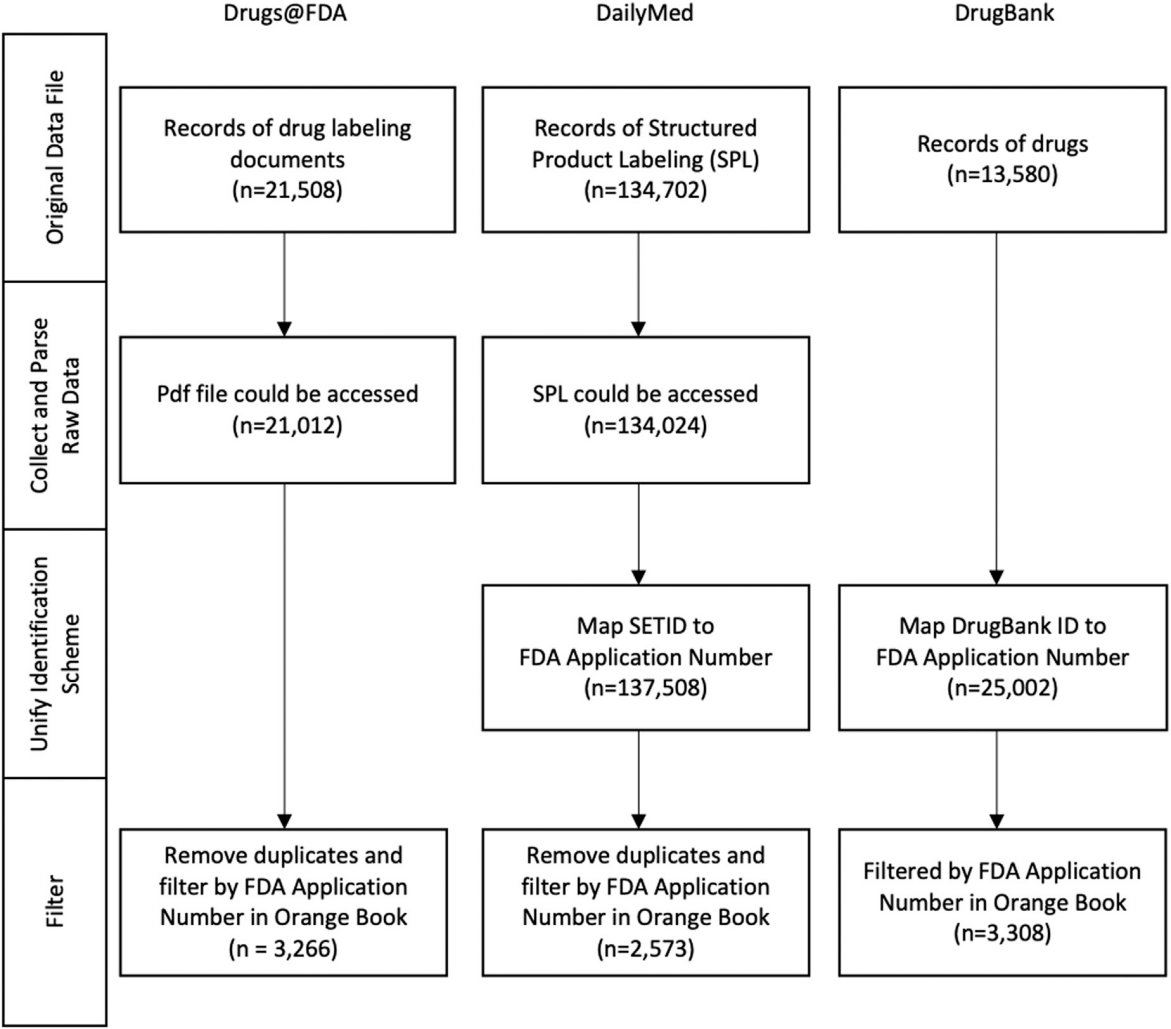
The birds’ eye view of the workflow used for information extraction from various data sources, as shown at the top, which includes Drugs@FDA, DailyMed and DrugBank. On the left depicts the main steps of information extraction. Note that the Orange Book is used as a point of reference for the FDA NDA (New Drug Application) application number with which the data extracted from multiple sources are integrated at the step of Filter (Downloaded Version: Oct. 2020).

The workflow applied to the aforementioned data sources for processing, i.e., Drugs@FDA, DailyMed and DrugBank, is summarized in Figure 1. It contained four steps in general, which need to be adjusted in a variety of ways to accommodate data source diversity. In this section, we illustrate how the workflow is implemented for each data source.

#### Data Collection and Parsing

Table 1 presented the details of the data format/access method, the download date/version and the number of records extracted from four different sources. In the original data files, Drugs@FDA and DailyMed provided the complete list to access the SPL and PDF files via RESTful API, whereas DrugBank provided the complete dataset in a single XML file.

**TABLE 1:** Data resources overview.

Data source	Data format/Access method	Version/Date	Number of records			
			Initial	Accessible	Unified ID	Filtered
Orange book	csv/datafiles	Oct. 2020	39,229	—	—	3,624
Drugs@FDA	Pdf/RESTful API	Nov. 24, 2020	21,508	21,012	—	3,266
DailyMed	Xml/RESTful API	Nov. 27, 2020	134,702	134,024	137,508	2,573
DrugBank	Xml/datafiles	July 2, 2020	13,580	—	25,002	3,308

After pulling the raw data, they were parsed, extracted and annotated based on the 12 sections (For details, see Table 4 below) which are essential for PSG assessment, including boxed warning, indication, dosage and administration, pregnancy, lactation, mechanism of action, pharmacodynamics, and five subsections under pharmacokinetics: absorption, distribution, metabolism, excretion, and food effect.

Drugs@FDA provides the latest FDA-approved drug labeling and previously approved labeling in PDF format. After having converted the PDF file to text, we extracted the 12 sections from free text via regular expression, which is a sequence of characters that define a search pattern. We used regular expression to detect and locate keywords/subtitles, then extract the related sections from the free text converted from pdf files.

DailyMed maintains the labeling in a document markup standard approved by HL7 referred to as SPL, which specifies various drug labeling sections by LOINC codes and names. Table 2 listed a sample of eight sections we collected in this paper. However, since absorption, distribution, metabolism, excretion, and food effect do not have LOINC codes, we extracted their parent section pharmacokinetics via LOINC codes first, then applied keywords detection and regular expression to locate subsections and automatically annotated them.

**TABLE 2:** LOINC of selected sections from drug labeling in DailyMed.

LOINC code	LOINC name
34066-1	BOXED WARNING SECTION
34067-9	INDICATIONS and USAGE SECTION
34068-7	DOSAGE and ADMINISTRATION SECTION
42228-7	PREGNANCY SECTION
77290-5	LACTATION SECTION
43679-0	MECHANISM OF ACTION SECTION
43681-6	PHARMACODYNAMICS SECTION
43682-4	PHARMACOKINETICS SECTION

DrugBank provides the complete dataset in a single XML format. Drugs are represented by <drug> tag. Its children elements contain both pharmacology information and commercial drug products. We extracted their contents from eight elements by tag names, including ADMEF information and the FDA application number whenever it is available. With the well-defined tags, DrugBank is a good complement when the subtitles of ADMEF are not available in Drugs@FDA or DailyMed.

#### Unify Identification Scheme

Different data sources have their own identification schemes. They vary from source to source. For example, the sources developed by the FDA such as Orange Book and Drugs@FDA use the FDA application number, a unique six-digit number to identify the drug, whereas DailyMed uses a unique Set ID to detect the SPL file. DrugBank uses its own DrugBank-ID for drug identification.

**FIGURE 2:**
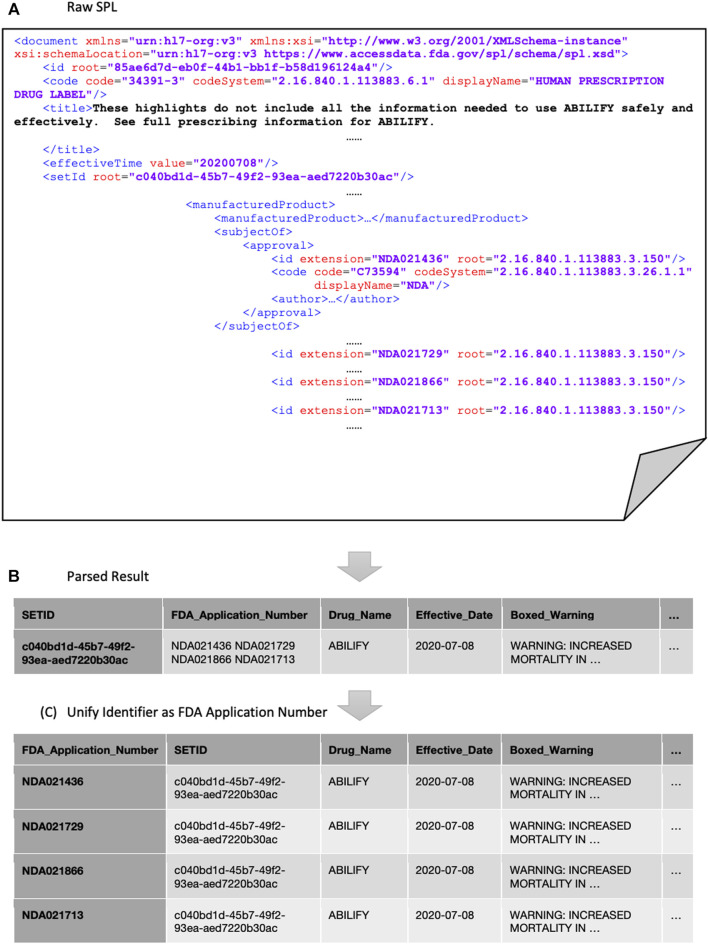
Unify identification scheme example (DailyMed SETID: c040bd1d-45b7–49f2-93ea-aed7220b30ac).

In order to provide a unified identifier to retrieve and combine information from multiple data sources, we mapped the Set ID in DailyMed and the DrugBank-ID in DrugBank to FDA application number, which is recognized by both Orange Book and Drugs@FDA. It is possible that one Set ID or DrugBank-ID may have more than one FDA application number. Under such circumstances, all the corresponding FDA application numbers are listed. Figure 2 illustrated a typical example as to how the Set ID was mapped in the parsed result of an SPL file to the FDA application number.

#### Filtering

Since both Drugs@FDA and DailyMed contain historical drug labeling, we only need to keep the latest version for each FDA drug application number with regard to the effective date of the latest document. Due to the different update schedules, each data source contains an inconsistent list of FDA-approved drugs. As the authoritative source of information in the United States on the therapeutic equivalence of FDA-approved drug products, the Orange Book contains 3,624 unique New Drug Application (NDA) FDA application numbers in the datafiles downloaded in Oct. 2020, which is used as a point of reference for the currently valid FDA-approved drugs in this paper.

### Paragraph Labeling of Food Effect

As described in the last section, we extracted information from drug labeling including Boxed Warning, Indication, Dosage and Administration, Use in Specific Populations, and Clinical Pharmacology. However, there are often complex scenarios (e.g., labeling food effect paragraphs from pharmacokinetics sections) where machine learning models are essential to achieve accurate information extraction. As such, we provide a case study for labeling food effect paragraphs as an example to illustrate how we address the scenarios when keyword detection and regular expression are not applicable.

**FIGURE 3:**
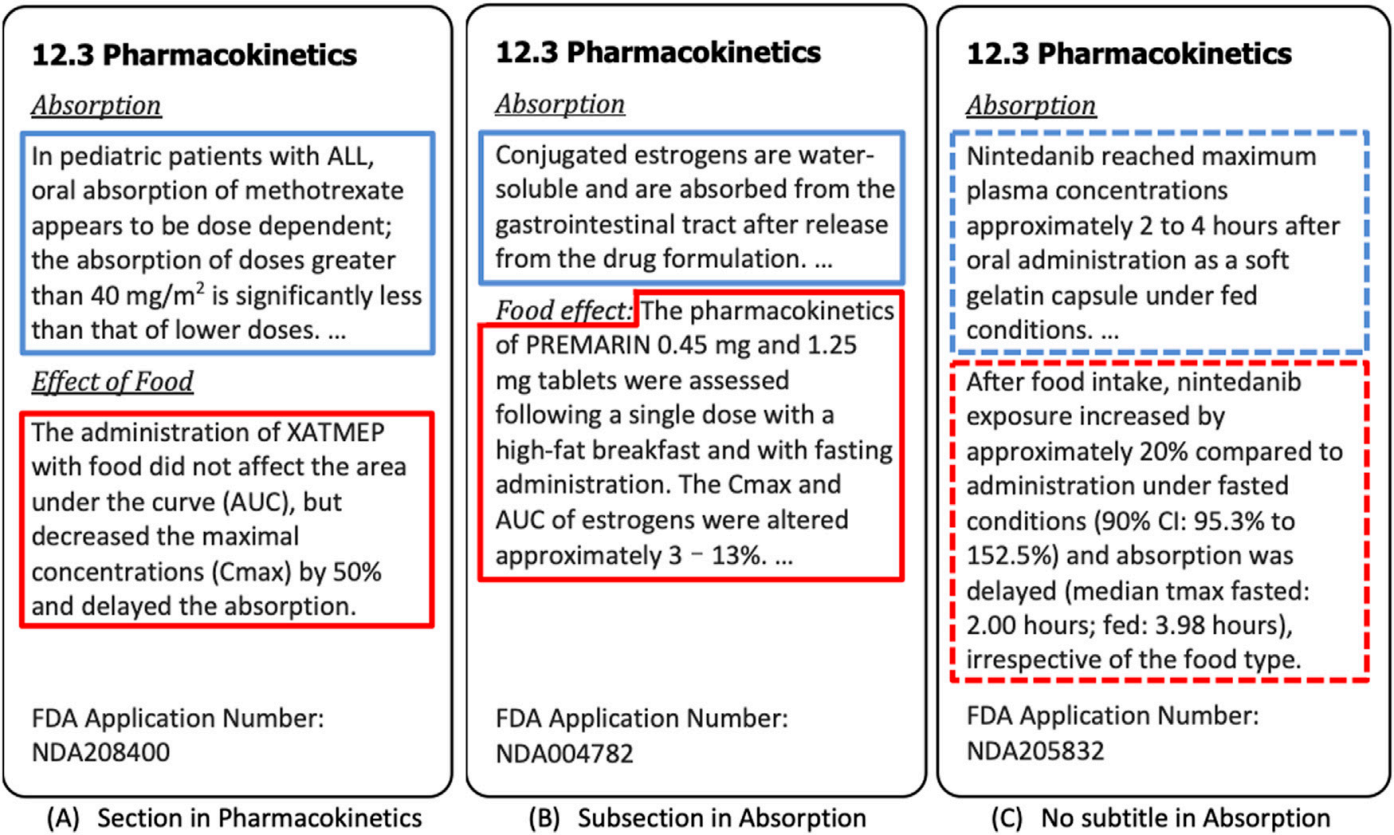
Three Examples of Food Effect Section. In **(A)** and **(B)**, the paragraphs in the blue border are annotated as “Non-Food Effect”, and the paragraphs in the red border are annotated as “Food Effect”. In **(C)**, the Food Effect paragraph classification model is able to identify the paragraph in the blue border as “Non-Food Effect”, and the paragraphs in the red border as “Food Effect”. The dashed borders signify the paragraphs labeled by the model, whereas the borders in solid line represent the paragraphs automatically labeled by using regular expressions.

Food may affect pharmacokinetics by any or all of the following mechanisms: delaying gastric emptying, stimulating bile flow, changing the pH of the gastrointestinal tract, increasing splanchnic blood flow, changing luminal metabolism of a drug, and physically/chemically interacting with a dosage form or drug (Sharma et al., 2013). Food effect paragraphs can appear in different sections in drug labeling. Figures 3A,B, respectively, provide examples of the subsection under pharmacokinetics and the subsection under the absorption section, where each paragraph has been automatically annotated as either “Food Effect” (red border) or “Non-Food Effect” (blue border). There are also the cases where the food effect paragraphs included in the absorption section without any subtitle (Figure 3C), which cannot be automatically extracted and easily annotated by keyword detection and regular expression. In these cases, the content of drug labeling relevant to the food effect is determined by semantic meaning, which motivated us to treat the food effect paragraph labeling as classification task (Food Effect vs. Non-Food Effect). We note that paragraph labeling is only one component (related to food effect) of our drug labeling extraction pipeline which integrates multiple disparate publicly available data resources to extract drug product information for enhancing PSG development.

#### Dataset

Food effect information is available in both Drugs@FDA and DailyMed. Table 3 shows the statistical summary of food effect sections we extracted from two data sources, where the numbers in the table represent the number of paragraphs. To train the model for identifying the food effect paragraphs from the absorption sections (e.g., Figure 3C), we constructed a dataset in which each paragraph was labeled as either Food Effect or Non-Food Effect. For Figure 3A, we detected the section title with regular expression “*^(food effect|food effects|effect of food|effects of food)$*” and annotated the paragraph that followed the title as “Food Effect”. For 3B, we used a slightly different regular expression “^(food effect|food effects|effect of food|effects of food)\s*(:*|-)*”, to detect if a paragraph belongs to “Food Effect”. We removed the title at the beginning of the paragraph that was detected by regular expression in the dataset. For other paragraphs under the section title detected by regular expression *“^absorption$*“, they were labeled as “Non-Food Effect”. The annotated results were manually checked afterwards to verify the paragraph labeling. Note that there was no change in the annotated label after manual checking. For model training and evaluation, we randomly selected 2,400 records in total (1,200 records from Drugs@FDA, 1,200 records from DailyMed), including both Food Effect and Non-Food Effect. Note that we used the equal number of records from Drugs@FDA and DailyMed (1,200 records each) to keep the data balanced and avoid potential bias during the training.

**TABLE 3:** Statistical Summary of Food Effect/Non-Food Effect Data Extracted by Keyword Detection and Regular Expression. The numbers represent the number of paragraphs.

	Non-food effect	Food effect		
		Food effect in pharmacokinetics section	Food effect in absorption section	Total
DailyMed	3,691	822	108	930
Drugs@FDA	1,166	327	74	401

#### Rule-Based Methods

We performed two rule-based methods as a baseline to compare the performance of machine learning models in test dataset. For the Rule-Based Method 1, if a paragraph matched the regular expression *“(food effect|food effects|effect of food|effects of food)*” anywhere, it was labelled as “Food Effect”; otherwise, it was labelled as “Non-Food Effect”. For the Rule-Based Method 2, we used a simple keyword search for “food” for paragraph labelling.

#### Machine Learning Models

Machine learning algorithms have been widely used for natural language processing, in which the text is represented by word embedding. Among various word embedding techniques, Term Frequency-Inverse Document Frequency (TF-IDF) perhaps is the most used statistical measure that evaluates how relevant a word is to a document (Chowdhury 2010). TF-IDF for a word in a document is calculated by multiplying two different metrics: Term Frequency (TF) and Inverse Document Frequency (IDF). TF-IDF score for the word *t* in the document *d* from the document set *D* is calculated as follows:

 TF-IDF (t,d,D)=TF(t, d)⋅IDF(t,D)

Where:

TF(t,d)=log[1+freq(t,D)],

$$IDF\left(t,D\right)=log\left(\frac{N}{count\left(d\in D:t\in d\right)}\right),$$

*N* refers to the total number of documents in the corpus and count(d∈D:t∈d) corresponds to the number of documents where the term *t* appears. The TF-IDF score is then fed to machine learning algorithms such as Logistic Regression and Support Vector Machines, which has been shown improved results over the basic word embedding methods such as Bag-of-Words (BoW). However, the methods based on the concurrence of terms, including the BoW and TF-IDF (that embeds the text by term frequency in the document, which lose the information about the relationship between the words), fail to identify syntactic and semantic relationships between words in the documents.

Language model pretraining has recently advanced the state of the art in many NLP tasks ranging from sentiment analysis, to question answering, natural language inference, named entity recognition, and textual similarity. State-of-the-art pre-trained models include ELMo (Peters et al., 2018), GPT (Radford et al., 2019) and more recently Bidirectional Encoder Representations from Transformers (BERT; Devlin et al., 2019). BERT combines both word and sentence representations in a single very large Transformer (Vaswani et al., 2017); it is pre-trained on vast amounts of text, with an unsupervised objective of masked language modeling and next-sentence prediction and can be fine-tuned with various task-specific objectives. With the pre-trained model, fine-tuning allows BERT to model many downstream tasks, such as text classification. While BERT has dramatically improved outcomes in NLP tasks in the general domain such as optimizing search results, its performance in domain-specific tasks such as drug labeling has not fully been explored.

In this paper, we assessed the predictive performance of three pre-trained BERT-base models: BERT, RoBERTa (Liu et al., 2019), and DistilBERT (Sanh et al., 2020). We compared these state-of-the-art approaches with three traditional machine learning algorithms: Logistic Regression, Linear Support Vector Classification (SVC), and Random Forest, with the TF-IDF as the word embedding method. We note that the input to the model was a single paragraph and the output was a label for that paragraph to indicate whether it was “Food Effect” or “Non-Food Effect”.

The BERT-base models were implemented by Simple Transformers library^7^. We used Adam optimizer (Kingma and Jimmy, 2017) with the learning rate of 4e-5. The train batch size was set to 32. To avoid exploding gradients, we clipped the gradients to the maximum norm of one. Other hyperparameters of the models were initialized by default values.

#### Evaluation

We evaluated the classification results from two perspectives: the impact of training and testing dataset and the performance of text classification algorithms.

To check the impact of the training and testing dataset on classification results, we created three datasets related to the data sources: Drugs@FDA (1,200 records), DailyMed (1,200 records) and the combined Drugs@FDA and DailyMed (Drugs@FDA + DailyMed, 2,400 records). For each dataset, we kept 80 percent data for training and the remaining 20 percent for testing. Then, we trained and tested on various combinations of datasets. For example, we trained the model by combined Drugs@FDA and DailyMed dataset (Drugs@FDA +DailyMed) and tested with the dataset only from DailyMed or trained the model by the dataset only from Drugs@FDA and tested with the dataset only from DailyMed.

To assess the performance of different machine learning algorithms for text classification, we used the Drugs@FDA and DailyMed and combined dataset. We used precision, recall and F1-score as the performance metrics, which are calculated by using the number of true positives (TP), false positives (FP) and false negatives (FN) as follows:

$$precision=\frac{TP}{TP+FP}$$

$$recall=\frac{TP}{TP+FN}$$

$$F1\mathrm{-s}core=2\cdot \frac{precision\cdot recall}{precision+recall}$$

## Results

### Information Extraction

There were total 12 sections (See Table 4 for details) in the drug product information we extracted in this work, only eight sections could be extracted by the LOINC codes (See Table 2 for details; we note that only DailyMed has the LOINC codes). For sections without the LOINC codes in DailyMed and all 12 sections in Drugs@FDA, we used regular expression to extract the data we needed (Data in DrugBank is an xml file, all sections of which are well defined by tag name). To identify food effect information, machine learning models such as DistilBERT were used to label food effect paragraphs in the absorption section.

**TABLE 4:** Comparison of Source Coverage and Overlap of Unique Drug and Drug Labeling Sections.

	Coverage (%)			Overlap (%)		
Drug labeling section	Drugs@FDA	DailyMed	DrugBank	Drugs@FDA	DailyMed	DrugBank
Boxed warning	83.01	77.79	—	36.62	39.08	—
Indication	56.97	75.01	96.49	89.07	80.75	66.04
Dosage and admin	67.85	88.97	—	41.87	31.93	—
Use in specific populations						
Pregnancy	74.44	85.07	—	39.97	34.97	—
Lactation	94.56	68.52	—	33.35	46.03	—
Clinical pharmacology						
Mechanism of action	56.23	51.64	96.93	86.67	91.46	53.47
Pharmacodynamics	43.38	38.04	95.51	84.36	91.10	38.97
Pharmacokinetics						
Absorption	41.48	34.33	96.68	82.69	91.26	36.63
Food effect	92.35	63.14	—	30.04	43.94	—
Distribution	53.05	44.09	88.28	76.87	85.33	44.28
Metabolism	47.92	38.73	96.07	81.26	90.97	42.26
Excretion	49.43	41.36	93.97	81.10	89.30	43.29
Drug	90.12	71.00	91.28	82.00	95.96	82.89

The coverage represents the percentages are relative to total counts of 3,624 unique drug, 977 unique boxed warning section, 3,414 unique indication section, 2,883 unique dosage and administration section, 2,578 unique pregnancy section, 1,655 unique lactation section, 3,356 of mechanism of action section, 3,294 pharmacodynamics section, and 3,134 absorption section, 510 food effect section, 2,722 distribution section, 3,101 metabolism section, 3,051 excretion section parsed from pharmacokinetics section. The overlap represents the percentages of the average number of sources sharing each labeling section.

The Orange Book data file downloaded in Oct. 2020 provided 3,624 unique drugs with FDA NDA application number, which provided the reference list of valid NDA application numbers in this paper.

Table 4 summarized how much the data was covered by each of the three other sources. DrugBank covered the largest number of unique drugs (91.28%), followed by Drugs@FDA (90.12%). For the drug labeling sections, where the information is available in DrugBank (indication, mechanism of action, pharmacodynamics, absorption, distribution, metabolism, excretion), it also has the largest coverage among the three sources. Drugs@FDA and DailyMed both provided more labeling sections than DrugBank, though Drugs@FDA has higher coverage than DailyMed in general.

To quantify the uniqueness of each source’s contribution, we computed the overlap measure, which is defined as the percentage of the average number of sources that contributed to each drug labeling section to the total number of the data sources. For example, 0% overlap indicates that the data source is the only source provided information for that drug labeling section, which has no overlap with other sources. Similarly, 100% overlap indicates that the information of the drug labeling section that one source provided completely shares with the other two sources. Drugs@FDA had the least overlap for the unique drugs (82.00%).

### Classification Performance of Food Effect by Machine Learning Models

Table 5 shows the results of the rule-based methods and different text classification models applied to the combined dataset of the Drugs@FDA and DailyMed. First, the machine learning models outperformed the ruled-based methods. Second, the BERT-based models performed better (with F1 score 0.9397, 0.9386 and 0.9607 for BERT, RoBERTa, and DistilBERT model, respectively) than traditional machine learning models (0.8889, 0.9004, and 0.9345 for Logistic Regression, Linear SVC, and Random Forest). The performance evaluation metrics reported are the average of ten random experiments. Random Forest and DistilBERT are the best performance model in traditional machine learning algorithms and BERT-base models, respectively. Next, we used these two models for further evaluation with different combinations of training and testing on various datasets.

**TABLE 5:** Result for rule-based methods and different machine learning models (Best model performance is in bold).

	Precision	Recall	F1
Rule-based method 1	0.6429	0.2348	0.3439
Rule-based method 2	0.5000	0.6522	0.5660
Logistic regression	0.9091	0.8696	0.8889
Linear SVC	0.8966	0.9043	0.9004
Random forest	0.9386	0.9304	0.9345
BERT	0.9316	0.9478	0.9397
RoBERTa	0.9469	0.9304	0.9386
DistilBERT	**0.9649**	**0.9565**	**0.9607**

Table 6 shows the different combinations of training and testing on various datasets. The results show that DistilBERT has a better performance than Random Forest. In general, both DistilBERT and Random Forest, trained on the Drugs@FDA and DailyMed, can improve the prediction performance on a single dataset alone, which indicates that the combined training dataset can help to overcome the differences between data sources. However, we observed one exception for DistilBERT that was trained with the combined data and tested with Drugs@FDA: though it did not reach the best performance, the F1 score (0.9313) is tied with that of Random Forest and the difference in F1 scores between different training datasets is rather narrow.

**TABLE 6:** Result with different combinations of training and testing on various datasets (Best model performance of each combination is in bold).

Train	Test	F1	
		Random forest	DistilBERT
Drugs@FDA + DailyMed	Drugs@FDA + DailyMed	**0.9345**	**0.9607**
DailyMed	Drugs@FDA + DailyMed	0.9204	0.9333
Drugs@FDA	Drugs@FDA + DailyMed	0.9067	0.9478
Drugs@FDA + DailyMed	DailyMed	**0.9388**	**0.9796**
DailyMed	DailyMed	0.9149	0.9388
Drugs@FDA	DailyMed	0.8866	0.9583
Drugs@FDA + DailyMed	Drugs@FDA	**0.9313**	0.9313
DailyMed	Drugs@FDA	0.9242	0.9302
Drugs@FDA	Drugs@FDA	0.9219	**0.9394**

To evaluate the model generalization ability to an unseen (but related) data, we tested both DistilBERT and Random Forest with a manually created manually-labeled dataset containing 64 “Food Effect” paragraphs and 46 “Non-Food Effect” paragraphs that are not detectable by regular expression. We can observe that the DistilBERT (precision: 0.9524, recall: 0.9375 and F1: 0.9449) also performed better than Random Forest (precision: 0.9474, recall: 0.8438 and F1: 0.8926) on these data.

**FIGURE 4:**
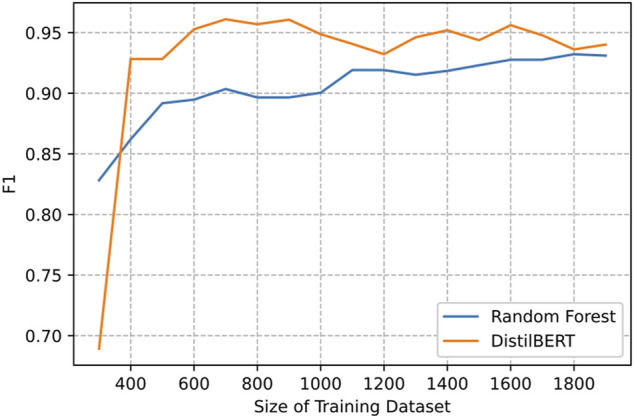
Learning curve of test performance with increasing amounts of training data.

To further substantiate that the pre-trained BERT model performs better than traditional machine learning methods, we show in Figure 4 by comparing the learning curves of the Random Forest with DistilBERT model when the training data were gradually increased. We observed that the DistilBERT reached an F1-measure > 0.9 even with a much smaller training dataset than Random Forest. Together, it is shown that the pre-trained BERT-based model is able to outperform the traditional machine learning techniques for food effect labeling on drug labeling datasets.

## Discussion and Conclusion

In this paper, we developed an NLP pipeline to perform information extraction from FDA drug labeling for PSG development. We integrated drug product information from multiple data sources (Orange Book, Drugs@FDA, DailyMed, and DrugBank) and have collected information with a variety of data formats via different access methods and addressed the inconsistent issue of the identification scheme of different data sources, which allows one to request the FDA-approved drug data by a unified identifier. Among these data sources, Drugs@FDA has relatively high coverage and the least overlap with other data sources, making itself ideally the primary source for the PSG assessment. Nonetheless, other data sources can still provide complementary information to Drugs@FDA for a comprehensive assessment of the drug products.

We used food effect paragraph labeling as an example to address the complex scenarios when keyword detection and regular expression are not adequate. For example, some “Non-Food Effect” paragraphs may include a sentence such as “Effect of food on the bioavailability of methadone has not been evaluated,” which matches the regular expression but is not related to food effect study of the drug. Similarly, some “Food Effect” paragraphs may contain the food effect content but without the keyword “food” (e.g., “A high-fat meal increased the extent and rate of naloxegol absorption. The Cmax and AUC were increased by approximately 30 and 45%, respectively. In clinical trials, naloxegol was dosed on an empty stomach approximately 1 h prior to the first meal in the morning.”). We further demonstrated the pre-trained BERT-based model, when fine-tuned on a small set of annotated labels, is able to outperform the traditional machine learning algorithms in text classification for food effect labeling on drug labeling datasets. This result was confirmed by checking the model on unseen test domain. For example, the second paragraph in Figure 3C was correctly identified as “Food Effect” by the BERT, yet completely missed by keywords/regular expression.

Several limitations need to be noted. First, we only apply this classifier to separate food effect paragraphs within the absorption section. As a result, the Non-Food Effect data only contains absorption data from drug labeling. Second, the minimum text unit we extracted and annotated is a paragraph. The food effect is not required to be written in a separate paragraph in the drug labeling and thus has the possibility to be either mentioned in a sentence within other sections or spanned more than one paragraph. In either case, it is possible that a small amount of food effect paragraphs can be mixed with the Non-Food Effect data. Third, when the size of the training dataset is limited, e.g., less than 400 as shown in Figure 4, Random Forest has a better performance than DistilBERT. As the amount of the data increases, the advantage of the BERT-based model becomes clear. Our observation is consistent with what is well known in the deep learning field that a deep learning model requires much more data than a traditional machine learning algorithm.

In summary, we developed a pipeline to integrate four publicly available data sources for drug product information various data formats and access methods. 12 related sections in the drug labeling were extracted from these data sources, which aimed to enhance the assessment process for PSG development. In addition, we demonstrated that the pre-trained BERT model is able to outperform the traditional machine learning techniques, setting a new state-of-the-art on drug labeling datasets to address the classification challenge for labeling food effect paragraphs, which can be adapted to other drug labeling sections or data sources.

## Data Availability

The original contributions presented in the study are included in the article/Supplementary Material, further inquiries can be directed to the corresponding author.

## Footnotes

1
https://www.accessdata.fda.gov/scripts/cder/psg/index.cfm

2
https://www.fda.gov/media/101052/download

3
https://www.accessdata.fda.gov/scripts/cder/ob/index.cfm

4
https://www.accessdata.fda.gov/scripts/cder/daf

5
https://dailymed.nlm.nih.gov/dailymed/

6
https://go.drugbank.com

7
https://github.com/ThilinaRajapakse/simpletransformers
